# Perspective view of allogeneic IgG tumor immunotherapy

**DOI:** 10.1186/s12935-024-03290-9

**Published:** 2024-03-09

**Authors:** Ying Liu, Yuanyi Huang, Hong-Wei Cui, YingYing Wang, ZhaoWu Ma, Ying Xiang, Hong-Yi Xin, Jun-Qing Liang, Hong-Wu Xin

**Affiliations:** 1 Department of Radiology, Jingzhou Hospital Affiliated to Yangtze University, Jingzhou, 434000 Hubei China; 2 Laboratory of Oncology, School of Basic Medicine, Center for Molecular Medicine, Health Science Center, Yangtze University, Jingzhou, 434023 Hubei China; 3 Department of Biochemistry and Molecular Biology, School of Basic Medicine, Health Science Center, Yangtze University, Jingzhou, 434023 Hubei China; 4 Center for Breast Cancer, Peking University Cancer Hospital at Inner Mongolia Campus and Affiliated Cancer Hospital of Inner Mongolia Medical University, Hohhot, 010021 Inner Mongolia China; 5 Division of Life Sciences and Medicine, Department of Obstetrics and Gynecology, Core Facility Center, The First Affiliated Hospital of USTC, University of Science and Technology of China, Hefei, Anhui China; 6 The Doctoral Scientific Research Center, People’s Hospital of Lianjiang, Guangdong, 524400 China; 7 The Doctoral Scientific Research Center, People’s Hospital of Lianjiang, Guangdong Medical University, Guangdong, 524400 China; 8 Key Laboratory of Human Genetic Diseases Research of Inner Mongolia, Research Centre of Molecular Medicine, Medical College of Chifeng University, Chifeng, 024000 Inner Mongolian Autonomous Region China

**Keywords:** Allogeneic IgG, FcγR, Dendritic cell, Antigen presentation, Tumor immunotherapy

## Abstract

Allogeneic tumors are eradicated by host immunity; however, it is unknown how it is initiated until the report in *Nature* by Yaron Carmi et al. in 2015. Currently, we know that allogeneic tumors are eradicated by allogeneic IgG via dendritic cells. AlloIgG combined with the dendritic cell stimuli tumor necrosis factor alpha and CD40L induced tumor eradication via the reported and our proposed potential signaling pathways. AlloIgG triggers systematic immune responses targeting multiple antigens, which is proposed to overcome current immunotherapy limitations. The promising perspectives of alloIgG immunotherapy would have advanced from mouse models to clinical trials; however, there are only 6 published articles thus far. Therefore, we hope this perspective view will provide an initiative to promote future discussion.

## Introduction

Immunotherapy has achieved durable responses in some cancer patients. However, the tumor microenvironment poses significant challenges that limit its effectiveness by creating an immunosuppressive milieu that shields tumors from antitumor immunity, thereby preventing many patients from benefiting from these therapies. Therefore, there is an urgent need to find new therapies to improve patient outcomes. As we reported in *the Journal of Hematology and Oncology* in 2020, dendritic cells (DCs) play a significant role in initiating and maintaining the immune response against cancer cells [1]. These specialized cells can recognize and process antigens, present them to T cells, and regulate immune responses upon uptake of immune complexes (ICs), making them attractive targets for cancer immunotherapy.

The Fc region of the antibody contains constant sequences, with only a small number of variants, and can elicit a host of cellular responses by binding to various Fc receptors expressed widely by different leukocytes. Fc–FcγR interactions represent a key component of the in vivo activity of therapeutic mAbs [2, 3]. The interaction of IgG–FcγR activates various downstream immune regulatory pathways with multiple functional consequences, including activation of DCs and T cells [4]. Carmi et al. found that allogeneic IgG (alloIgG) can combine with dendritic cells to induce a powerful T-cell antitumor response [5]. This review and perspective review introduces the effects and safety of alloIgG tumor immunotherapy and the underlying mechanisms for its potential future clinical application.

## Immunoglobulin G and FcγR are important in immunity

Immunoglobulin G (IgG) comprises 10–20% of all plasma proteins and 70–75% of total immunoglobulins [6]. Its high antigen affinity, somatic hypermutation, and essential role in immune memory are well established. IgG includes IgG1, IgG2, IgG3, and IgG4 subtypes. IgG1 and IgG3 have the highest affinity for type I Fcγ receptors (FcγRs) for increased cytotoxic activity in vivo*,* while IgG2 and IgG4 have poor affinity with all type I FcγRs [7]. In addition to activating C1q, IgG has multiple functions, including binding to FcγRs on immune cells.

When multimeric IgG immune complexes interact with activated FcγRs, receptor clustering and aggregation occur, leading to phosphorylation of the ITAM structural domain by SRC family kinases (such as LYN, LCK, HCK, and FGR) and the recruitment and activation of SYK family kinases [8, 9]. This event activates the PI3K-PKC pathway, resulting in Ca_2_^+^ mobilization and cellular activation [10, 11]. MEK and MAP family kinases and the Ras pathway are then activated [12]. The IgG–FcγR interaction and downstream signaling may lead to antibody-dependent cellular cytotoxicity (ADCC) or phagocytosis (ADCP), cytokine and chemokine release, leukocyte differentiation and survival and T/B-cell responses [13–15].

Furthermore, IgG can directly neutralize toxins and microbes [16]. IgG can also generate inflammatory mediators and eliminate opsonized microbes [17]. An IgG molecule contains two variable Fab domains for antigen binding, one constant Fc domain for FcγR binding and a hinge region in a Y shape [18]. Although the Fc domain has conventionally been considered the invariant domain of an IgG molecule, it exhibits remarkable structural heterogeneity with different IgG subclasses and biantennary N-linked glycans [19]. These structural determinants modulate the conformational flexibility of the IgG Fc domain and impact its ability to bind to different types of FcγRs (type I or type II) [20].

FcγRs are widely expressed on immune cells and specifically bind to the IgG Fc domain [21]. Activating FcγRs include FcγRI, FcγRIIA, FcγRIIIA, and FcγRIIIB (CD64, CD32a, CD16a, CD16b) in humans and FcγRI, FcγRIII, and FcγRIV in mice [7]. A single inhibitory receptor, FcγRIIB (also known as CD32b), is activated by a tyrosine inhibition motif (ITIM) in both humans and mice [22, 23]. Activating FcγR mRNA is expressed in monocytes, macrophages, and monocyte-derived DCs (moDCs), and inhibitory FcγRIIB mRNA is expressed in mouse cDCs, plasmacytoid DCs (pDCs), moDCs and macrophages. Human cDCs and pDCs express FcγRIIB mRNA as well as FcγRIIA. Both mouse and human CD172α^+^ cDCs express low levels of FcγRI, as determined by flow cytometry [13, 20]. Although mRNA expression does not always predict protein expression, recent human and mouse flow cytometry data support these findings [24]. These data suggest that macrophages and moDCs express mRNA for most of the activating and inhibitory FcγRs, whereas cDCs and pDCs primarily express mRNA for the inhibitory FcγRIIB.

The relative expression of activating and inhibiting FcγRs coexpressed on many immune cells determines the activation threshold of immune cell responses [25]. FcγRIIb activation leads to receptor cross-linking, phosphorylation by SRC family kinases and phosphatase recruitment to their ITIM structural domains [26, 27]. ITIM-recruited phosphatases (SHIP1 and SHP2) lead to the hydrolysis of phosphatidylinositol 3,4,5-triphosphate (PIP3) to phosphatidylinositol 4,5-biphosphate (PIP2), inhibiting PLCγ and the tyrosine kinase BTK [27–29].

## IgG mAbs tumor immunotherapy requires IgG Fc–DC FcγR interaction

Therapeutic monoclonal antibodies (mAbs) interact with innate and adaptive immunity in vivo [7]. Therapeutic mAbs bind to cancer cell surface antigens, inhibiting their proliferation and survival [30]. The IgG Fc–FcγR interaction mediates ADCC, ADCP, and CDC functions to block growth signals and angiogenesis and activate the immune response [7, 31]. Despite the diverse mechanisms of action of therapeutic mAbs, a common function is their interaction with FcγRs expressed on the surface of leukocytes through their Fc domain. FcγRIIIa significantly improves the therapeutic efficacy of anti-CD20 monoclonal antibodies [32]**.** B-cell lymphoma, breast cancer, and colorectal cancer patients carrying FcγRIIa and FcγRIIIa allelic variants are more responsive to antitumor antibody therapy [33–36]. In HER2^+^ breast cancer, mAbs have become the frontline standard of care, outperforming HER2-specific small molecule inhibitors and achieving excellent responses with modest toxicities [37], which require IgG–FcγR interaction [38]. Anti-GITR antibodies were found to require activating FcγRs [39]. Fc–FcγR interactions can promote innate immunity via cellular differentiation and survival. Fc–FcγR interactions promote antigen processing and presentation and the maturation and activation of dendritic cells [4]. Last, B cells are also regulated by Fc–FcγR interactions by their type I FcγR, FcγRIIb and type II FcγR, CD23 [40]. These results suggest that Fc–FcγR interactions are vital in cancer immunotherapy.

## IgG Fc–DC FCγR interaction activates DCs and T cells

**Fig. 1:**
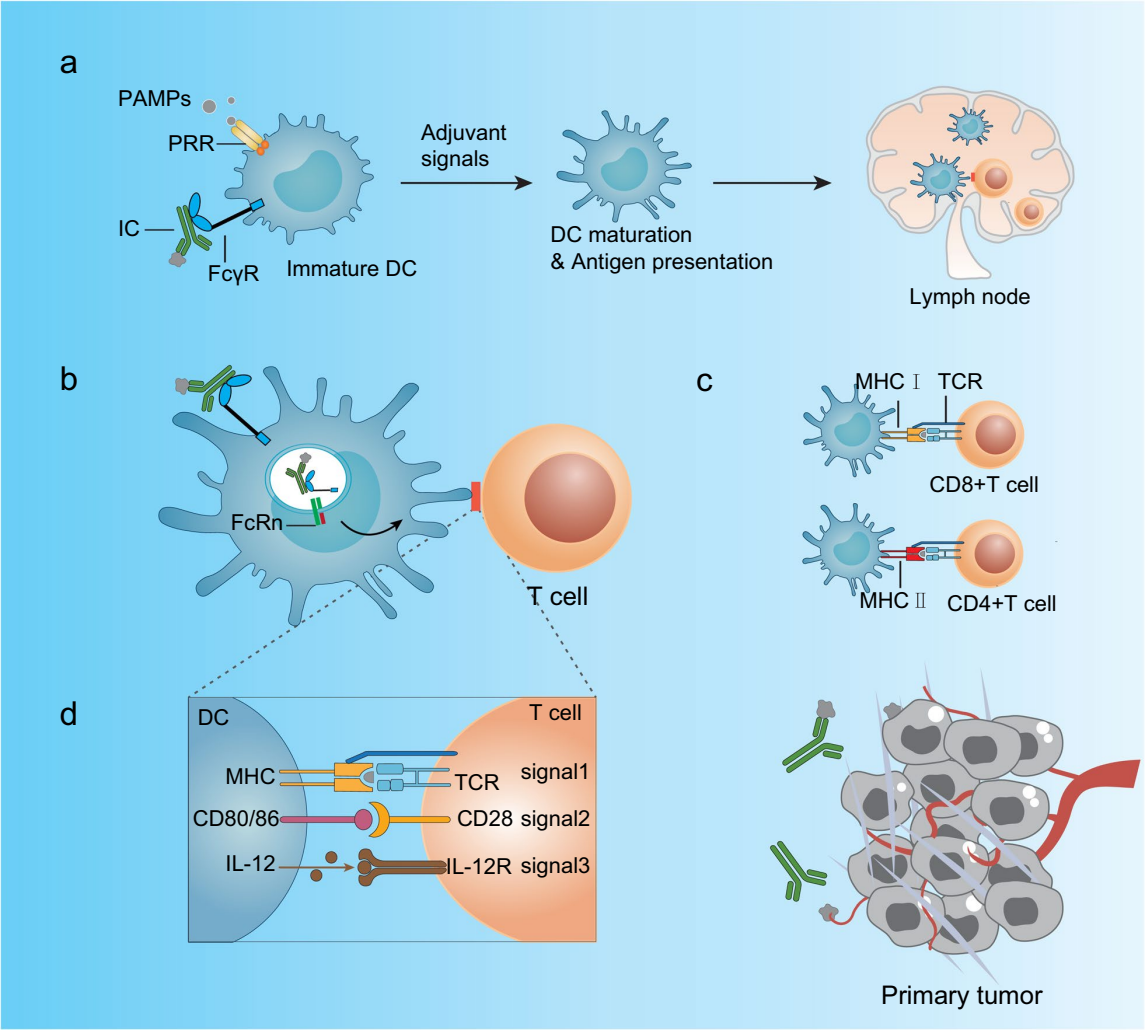
IC–FCγR interactions lead to DC antigen presentation and T-cell activation. **a** When exposed to foreign antigens, PRRS and FcγRs can mediate the induction of dendritic cell maturation. During maturation, imDCs lose their endocytic capacity while increasing their capacity for antigen processing and presentation, driving their homing to lymph nodes, where they present antigens to naive CD4+ or CD8+ T cells. **b** ICs bind to FcγRs on the surface of DCs, are internalized and processed, and subsequently bind to neonatal FcRn, trafficking and MHC-mediated antigen presentation. **c** Matching of TCR with MHC molecules on DCs. **d** The activation of a T-cell depends on its interaction with APCs and requires three signals

DCs are the most effective antigen-presenting cells (APCs) [41]. DCs include type 1 cDCs (cDC1s), type 2 cDCs (cDC2s), and pDCs. DCs become activated upon exposure to foreign antigens, which can occur through the engagement of conserved bacterial or viral antigens known as pathogen-associated molecular patterns (PAMPs) via pattern recognition receptors (PRRs) [42]. Resting immature DCs (imDCs) express PRRs of Toll-like receptors (TLRs), membrane-associated C-type lectin receptors, and mannose receptors [43–45]. DC maturation is regulated by activating and inhibitory type I FcγRs. Steady-state DCs express both inhibitory FcγRIIb and activating FcγRIIa, which prevents inappropriate or uncontrolled DC maturation [7]. Selective blockade of FcγRIIB using monoclonal antibodies leads to human DC maturation [46]. imDCs become mature, losing their endocytic capacity but increasing their antigen processing and presentation capacity [47]. maDCs upregulate chemokine receptors such as CCR7, driving their homing to lymph nodes [48], where they present antigens to naive CD4^+^ or CD8^+^ T cells (Fig. 1a) [49, 50]. cDC1s cross-present antigens to cytotoxic CD8^+^ T cells and promote the activation of CD4^+^ T helper type 1 (Th1) cells, while cDC2s induce CD4^+^ T-cell responses [51]. pDCs in the blood and spleen express MHC class II and costimulatory molecules [52]. Newly identified moDCs are present in mouse and human tumors [53]. MoDCs in tissues have a limited capacity to transport antigens to lymph nodes and activate naive T cells in vitro, which distinguishes them from Ly6C^+^ or CD14hi monocytes [54, 55]. Therefore, it is unclear to what extent moDCs contribute to the initiation of new T-cell responses.

Activated FcγRs promote degradative antigen processing and presentation, thereby activating T cells, while internalization by FcγRIIB tends to preserve the intact antigen for subsequent transfer to B cells. ICs are bound to FcγRs on the surface of DCs, internalized and subsequently bound to the neonatal Fc receptor (FcRn). Since FcRn is predominantly intracellular and binds IgG at acidic pH, it is well placed to engage IgG–ICs within endolysosomal compartments and regulate IgG–IC trafficking and MHC-mediated antigen presentation (Fig. 1b) [56]. ICs are more effective in antigen-presenting functions of DCs than free antigens [56, 57]. In mouse studies, DCs from splenic mice showed more efficient uptake of ovalbumin (OVA) preincubated with anti-ovalbumin IgG than “naked” OVA. Notably, OVA:IgG immune complexes induced CD4^+^ and CD8^+^ T-cell proliferation more effectively than “naked” OVA in mice transplanted with OVA-specific CD8^+^ or CD4^+^ T cells [58]. Inactivated *Francisella tularensis* immune complexes (mAb-iFt) are a more protective vaccine against lethal tularemia than iFt alone. Nelson et al. discovered that targeting iFt to FcγRs via mAb-iFt leads to enhanced DC maturation, with FcγR being needed for mAb-iFt-induced maturation of bone marrow-derived DCs [59]. Fc–FcγR interactions hold promise for DC-specific vaccination-based strategies [7, 60].

The activation of a T-cell depends on its interaction with APCs and requires three signals (Fig. 1b–d). Signal 1 is the specific peptide recognized by the T-cell receptor (TCR). Major histocompatibility complex (MHC) molecules (either MHC-I for cytotoxic CD8^+^ T cells or MHC-II for CD4^+^ T cells) are needed (Fig. 1c) [61]. The intracellular pathways [1, 50] mediate antigen degradation and peptide loading onto MHC molecules. Antigen presentation to CD4^+^ T cells is enhanced only when the antigen and IgG are present within the same phagosome [24]. Signal 2 is the costimulatory molecules on T cells (e.g., CD28) and their receptors on APCs, such as CD80 and CD86 (B7.1 and B7.2). Signal 3 is the cytokines needed to define the type of response. These three signals induce Ag-specific CD4^+^ or CD8^+^ T-cell responses (Fig. 1d) [62]. DCs can ingest virus-infected or tumor cells and present Ags to specific CD8^+^ T cells via cross-presentation through an MHC-I pathway [63].

## Tumors are eradicated by allogeneic IgG via DCs

The combination of tumor-binding alloIgG and DCs has been shown to effectively eradicate both primary and metastatic mouse tumors, including melanoma, pancreatic, lung, colon, and breast cancer (Table 1) [5]. In syngeneic C57BL/6 mice, B16 melanoma cells proliferated, while they were rejected in allogeneic 129S1 mice, with all animals treated by other methods experiencing rapid tumor recurrence. Allogeneic transplanted tumors had more mature myeloid DCs that were more activated than syngeneic tumors. IgM and IgG antibodies binding to allogeneic tumors enabled tumor-infiltrating DCs to process and present tumor antigens to CD4^+^ T cells, and this response was abrogated in FcγR-deficient mice. Only allogeneic immunoglobulin-IC could activate bone marrow-derived DCs (BMDCs) in vitro, and BMDC activated by alloIgG-IC induced significant T cell proliferation [5]. However, only minor effects were observed when alloIgG was injected into tumors in autologous mice in vivo. The possible explanation of the limited effect could be the difference between BMDCs and tumor-associated dendritic cells (TADCs) as described below.

**Table 1:** AlloIgG tumor immunotherapy

Ref.	Antibody	Stimuli	Subject	Tumor	Administration	Result (% tumor free, tumor size mm^2^)	Conclusion
[5]	AlloIgG-IC+BMDC	No	Mouse/in vitro	B16 melanoma and LMP pancreatic tumor	S.C Tumors removed upon reaching 25–55 mm^2^, leaving tumor-free margins	% tumor free LMP: 100% B16: > 40d, 75% (n = 5)	Injecting alloIgG-IC+BMDC protected naive mice from tumor challenge
	AlloIgG-IC	Poly(I:C), TNFα+CD40L or IFNγ+D40L	Mouse*/*in vitro	B16 and LL/2 tumors	Intratumoral injection	Tumor size: AlloIgG-IC+TNFα+CD40L: B16 < 8d 20 → 0 (n = 6); LL/2 < 10d 20 → 0 (n = 8) alloIgG-IC+Poly(I:C): B16 < 8d 20 → 7 (n = 6)	PolyI:C, TNFα+CD40L or IFNγ+CD40L enabled activation of TADC and alloIgG-IC uptake
	Crosslinked-synIgG-IC+BMDC	No	Mouse/in vitro	B16	Crosslinked synIgG onto B16 membrane proteins and incubation with BMDC	% tumor free: 80% (n = 8)	Binding of IgG to the tumor cell surface, rather than the origin of the IgG, was critical
	Anti-GP-NMB	TNFα+CD40L	Mouse/in vitro	B16	Intratumoral injection	Tumor size (mm^2^): < 15d 20 → 20 (n = 8)	Anti-GP-NMB+αCD40+TNFα induced significant FcγR-dependent tumor regression
	AlloIgG-IC	TNFα+CD40L	Mouse/in vitro	Metastases and primary 4T1 breast tumors		Tumor number: 0 (n = 4) Primary tumor size: 0 (n = 5)	AlloIgG+αCD40+TNFα led to almost complete resolution of metastases and primary tumors
	AlloIgG-IC	TNFα+CD40L	Human/in vivo	Malignant pleural mesothelioma			Drive the proliferation and activation of autologous CD4+ T cells
[96]	Anti-TRP1 antibody	TNFα+CD40L	Mouse/in vitro	B16F10/ret transgenic mice	Allowed B16F10 to grow until they reached a palpable size. Then, intratumoral injection	Tumor size (mm^2^): Treated on < 12d or tumor smaller than < 20 mm^2^: tumor regression > 12d: inert	TNFα+anti-CD40+anti-TRP1 antibody fails to eradicate late-stage melanoma tumors (n = 4)
[64]	AlloIgG-IC+BMDC	No	Mouse/in vitro	B16F10/LMP	Tumors reached 20–25 mm^2^ in size then surgically removed, leaving margins of approximately 1 mm	Tumor free: LMP: 100% B16: > 40d, 60% (n = 10 for control group and n = 5 in each treatment)	AlloIgG-IC-loaded BMDC prevent tumor recurrence following resection
	AlloIgG-IC+TADC/MoDC	SHP-1/2 inhibitor + ionomycin	Mouse/in vitro	B16F10	MoDC&TADC cultured overnight with alloIgG-IC alone or with SHP-1/2 inhibitor + ionomycin, then s.c. injected naive mice. B16 cells challenged on d5	Tumor free: AlloIgG-IC+MoDC+SHP-1/2inhib + ionomycin:100% AlloIgG-IC+TADC+SHP-1/2inhib + ionomycin: > 15d: 75% (n = 10 control, n = 5 test)	Simultaneous blockade of SHP-1 and phosphatases regulating Akt enables tumor and MoDC activation to facilitate tumor rejection
[87]	AlloIgG-IC	IFNγ+CD40L	Mouse/in vitro	MMTV-PyMT triple-neg. breast cancer	Tumors grew to 25 mm^2^, intratumoral injection	Tumor size (mm^2^): 25 → 0	The effective tumor-binding antibody therapy activates dendritic cells, which can prime T cells in the periphery
[101]	Anti-TRP1 antibody	After 6 days of treatment with anti-TRP1+TNFα+CD40L, CD4+ and CD8+ T cells isolated from the tumors, blood, and DLN	Mouse/in vitro	B16 melanoma	CD4+ or CD8+ T cells with or without anti-TRP1+ with or without DC stimuli injected i.v. into tumor-bearing mice	Tumor size (mm^2^): Anti-TRP1+TNFα+CD40L+CD4+ T cells: 25 → 20 Anti-TRP1+TNFα+CD40L+CD8+ T cells: 25 → 65	Adoptive transfer of CD4+ T cells, but not CD8+ T cells, induces potent tumor regression when combined with tumor-binding antibodies
	Anti-TRP1 antibody	TNFα+CD40L+PB or tumor or DLN CD4+ T cells	Mouse/in vitro	B16 melanoma	Anti-TRP1+TNFα+CD40L+PB or tumor or DLN CD4+ T cells injected i.v. into tumor-bearing mice	Tumor size (mm^2^): TNFα+CD40L+tumor or DLN CD4+ T cells: 25 → 10 TNFα+CD40L+PB CD4+ T cells: 25 → 100	CD4+ T cells from the tumor and DLN, but not from peripheral blood, directly kill tumor cells coated with IgG antibodies
[97]	Anti-TRP1 antibody	TNFα+CD40L	Mouse/in vitro	B16 melanoma		Tumors were completely eradicated in all mice. Nonetheless, after approximately 10d, half the mice developed recurrent tumors that were resistant to subsequent treatments	Cell-in-cell formation spatially prevents the penetration of T-cell-derived lytic granules to the inner tumor cells

Some results in the table were estimates from the graphs of the cited literature

*iv* intravenous injection, *sc* subcutaneous injection, *d* day, *IC* immune complex, *BMDC* bone marrow-derived dendritic cell, *TADC* tumor-associated dendritic cell, *MoDC* monocyte-derived dendritic cell, *DLN* draining lymph nodes, *PB* peripheral blood

## AlloIgG combined with DC stimuli TNFα and CD40L eradicated tumors

**Fig. 2:**
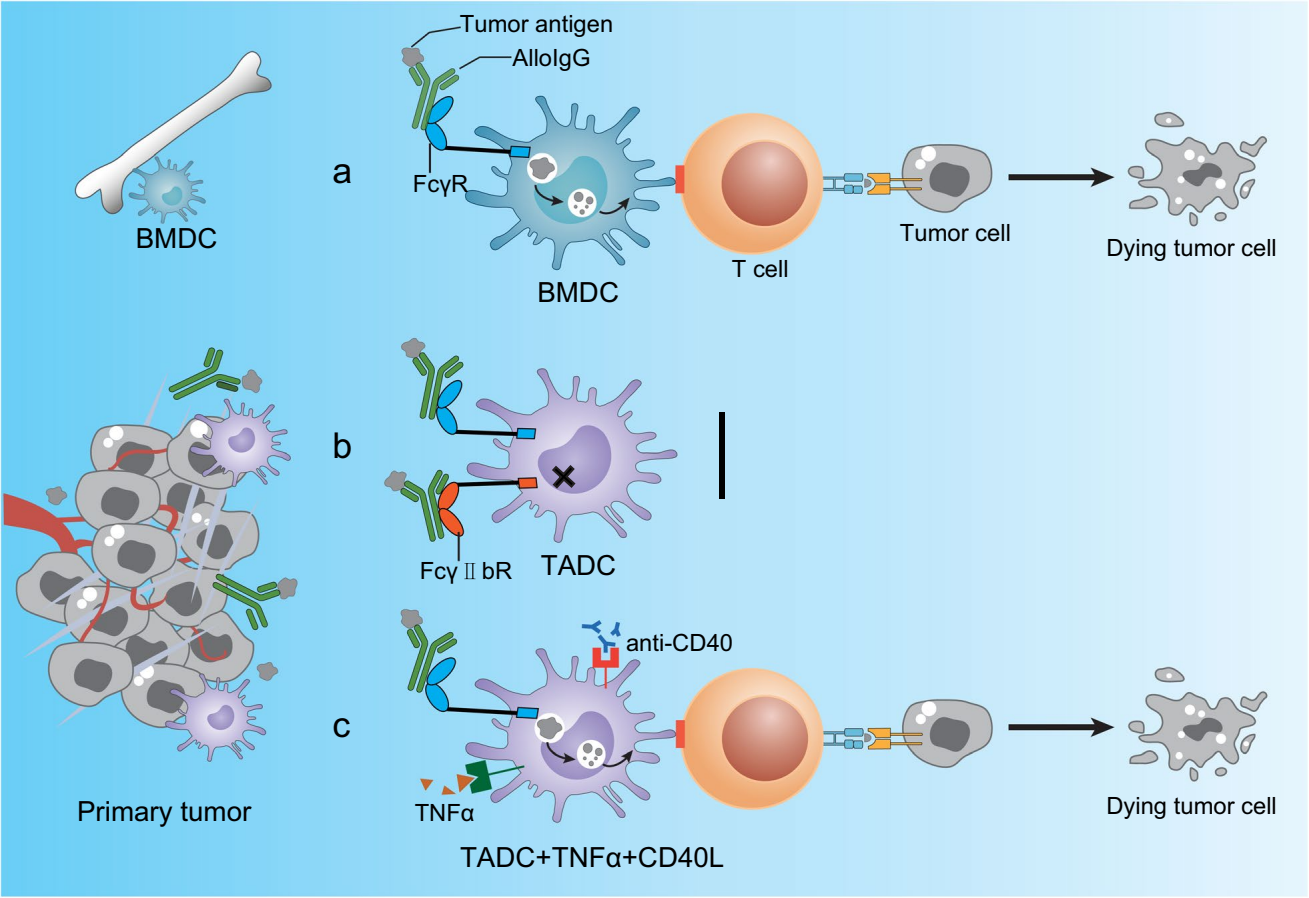
AlloIgG combined with TNFα and CD40L induced complete elimination of tumor cells. **a** Syngeneic BMDCs loaded with AlloIgG-IC activate T cells and prevent tumor recurrence in mice. **b** When AlloIgG was injected into tumors in autologous mice, TADC cannot transmit signals through their Fcγ receptor after contact with AlloIgG-IC in a highly immunosuppressed tumor microenvironment. **c** Combining tumor-binding AlloIgG with TNFα and CD40L enables TADC to internalize tumor antigens via the Fcγ receptor. These antigens are then processed by DCs and presented to T cells, which attack primary tumors and distant metastases

**Fig. 3:**
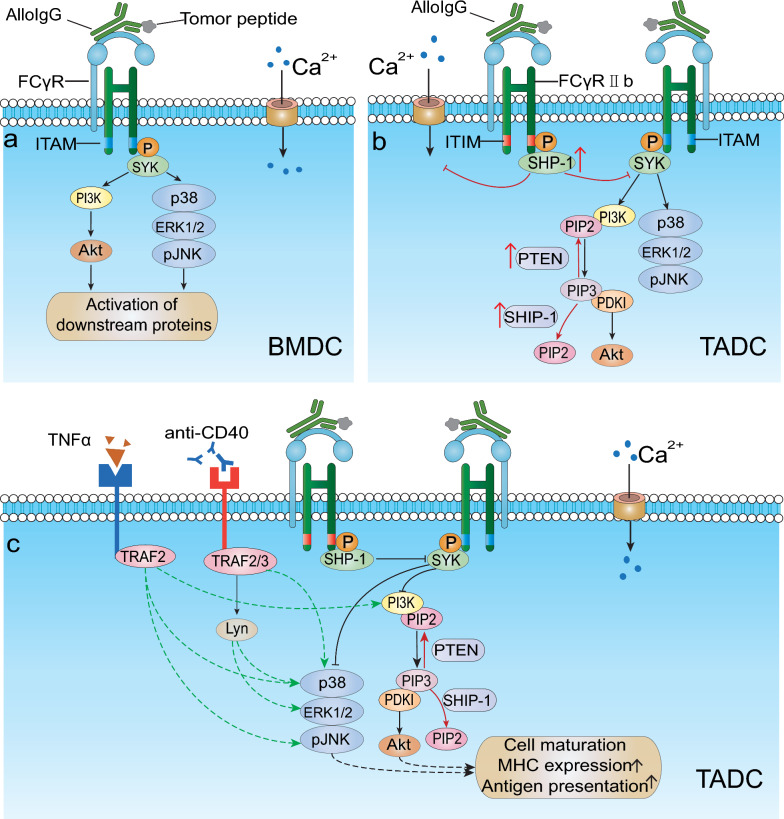
Mechanisms of DC-mediated signaling by alloIgG action. **a** Stimulation of BMDCs with AlloIgG-IC resulted in a significant increase in phosphorylated MAPK p38, ERK1/2, and JNK, as well as robust phosphorylation of Akt. **b** Once monocytes are released from their bone marrow niche into the circulation, they markedly elevate the levels of phosphorylated SHP-1 and phosphatases that regulate Akt activation. **c** Simultaneous blockade of SHP-1 and Akt-regulating phosphatases (such as PTEN and SHIP-1) enables activation of TADCs and MoDCs. Green arrows represent our proposed potential TADC/MoDC activation signaling pathways by TNFα and CD40L

Unlike BMDCs, TADCs did not respond to alloIgG against tumor cells or lysate (alloIgG-IC) (Fig. 2a, b). However, PolyI:C, TNFα^+^CD40L, or IFNγ^+^CD40L could activate TADCs to take up and present alloIgG-IC. Intratumoral injection of alloIgG combined with TNFα^+^CD40L or PolyI:C eliminated B16 and LL/2 homologous tumors. In mouse models, alloIgG^+^CD40^+^TNFα almost eliminated melanoma and breast cancer metastases (Fig. 2c). Culturing malignant pleural mesothelioma patients BMDCs with allogeneic IgG activated and enhanced autologous CD4^+^ T-cell propagation. Finally, the authors found that alloIgG isolated from healthy donors could similarly induce TADC activation in the presence of tumor necrosis factor alpha (TNF-α) and CD40 when cultured with tumor cells, validating the clinical performance of this approach. These results suggest that TADC unresponsiveness to IC is not due to the suppressive nature of the tumor microenvironment but rather a consequence of normal monocyte maturation [5]. Analysis of the signaling pathways in MoDC, TADCs, and BMDCs indicates that rapid Syk phosphorylation following ligation of FcγRs with ICs induces dramatic downstream protein activation in the MAPK (p38, pJNK, pERK) and PI3K/Akt (pAkt) pathways in BMDCs (Fig. 3a) [64]. Although SHP-1 regulates DC activation and Syk phosphorylation, inhibition of SHP-1 alone is not sufficient to induce MoDC or TADC to respond to IC. But it requires both PTEN and SHP-1/SHIP-1 for MoDCs and TADCs to activation by alloIgG-ICs (Fig. 3b) [64].

## Hypothetical potential signaling pathways in alloIgG-CD40-TNF-α-activated DCs

Next, we hypothetically propose the signaling pathways in alloIgG-CD40-TNF-α-activated DCs for the first time as a perspective view for further discussion (Fig. 3). We believe the mechanistic study will provide a basis for its future improvement. CD40, as a tumor necrosis factor, primes DCs for effective and specific T-cell activation [65]. Activation of DCs with CD40 agonists increased survival and cytokine secretion of IL-1, IL-6, IL-8, IL-12, TNF-α, and macrophage inflammatory protein-1α and upregulated costimulatory molecules of MHC class II, LFA-3, CD80, and CD86, promoting antigen presentation, priming, and cross-priming of T helper cells and cytotoxic T lymphocytes, respectively [66]. Based on their study, Vidalain and colleagues propose a model of CD40-mediated signaling in human DCs that includes CD40-induced membrane raft reorganization and the recruitment of TNFR-associated factors 2 and 3 (TRAFs) and activation of Lyn and other Src family kinases. Lyn activation leads to IL-1α, IL-1β, and IL-1Ra mRNA expression through a MEK/ERK pathway. Activation of p38 MAPK, which induces the expression of IL-12 mRNA, is likely stimulated through a TRAF-initiated pathway and, to some extent, through a Src family kinase-dependent pathway in the early phase of CD40 signaling [67]. Additionally, TRAF activates the NF-κB, MAPK, PI3K, and PLCγ pathways [68].

The essential role of CD40L in the induction of protective tumor immunity led researchers to expect that agonistic anti-CD40 antibodies would act as potent adjuvants to promote tumor immunity. CD40-stimulated DCs significantly induce T-cell proliferation and cytokine production [69]. CD40 engagement provides survival signals to DCs, making them resistant to Fas ligand expressed by activated T cells [70, 71]. CD40 agonistic antibodies generated CTL responses that eradicated lymphoma tumors. CD40 ligation could overcome peptide-induced peripheral CTL tolerance and increase antitumor efficacy [72–74]. Evidence suggests that the effect of anti-CD40 antibodies on CD40-expressing cells critically depends on whether they interact with FcγR and C1 [75]. Therefore, the CD40/CD40L interaction is necessary for optimal antigen presentation by DCs. However, some studies suggest the opposite result, indicating that CD40/CD40L may be closely associated with tumorigenesis [76]. CD40 is expressed on the surface of normal cells and cancer cells of the bladder, lung, and ovary [77–79] and is highly expressed in malignant hematological tumors [80]. CD40L is highly expressed in many cancers, but its tumorigenic functions in neoplastic disease remain controversial [81].

TNF-α is a potent anticancer cytokine that binds to two receptors, TNFRSF1A (TNFR1) in all cell types and TNFRSF1B (TNFR2) in immune cells. Activation of the NF-κB, JNK, p38 MAPK, ERK, and PI3K pathways by TNF-α binding to TNFR2 guides cell proliferation and survival [82] (Fig. 3c). Despite its multiple functions, TNFα can have conflicting effects on cancer cells. As demonstrated by Carswell, elevated levels of TNFα can eliminate MCA-induced sarcomas, and approximately 28% of cancers are sensitive to sTNFα [83]. Direct intratumor injection of DCs into homologous mouse tumors can reverse established tumor nodules in mice and provide effective immunity against subsequent tumor threats. This antitumor effect can be enhanced by prepriming DCs with recombinant TNF-α [84]. Cancer cell secretion of TNFα can promote DC production, differentiation, and maturation [85]. However, low levels of TNFα expression may be protumorigenic, as reviewed in detail by Balkwill [86].

## AlloIgG triggers systematic immune responses targeting multiple antigens

It has been found that alloIgG binding specifically to tumor cells, rather than the source of IgG or their cross-linking with syngeneic IgG, induces strong immune responses [5]. Syngeneic IgG bound only six B16 membrane proteins, but alloIgG preferentially bound 16 cell membrane proteins, including transmembrane glycoprotein NMB (GP-NMB) [5]. GP-NMB antibodies, αCD40 and TNFα together activate DCs and induce FcγR-dependent tumor regression with activated effector/memory T-cell infiltration, suggesting that tumor-reactive T cells targeting tumor-associated antigens that are not widely expressed alloantigens are needed [5].

It was shown that alloIgG triggers systematic immune responses [5]. Systematic immune responses were also reported in tumor-specific McAb IgG therapy. Spitzer, Matthew et al. developed an intuitive model, a computational method called scaffold maps. Scaffold map analysis revealed that treatment of a spontaneous model of carcinom MMTV-PyMT triple-negative breast cancer with anti-PD-1 antibodies triggered only a transient immune response at the local tumor, but the combination of tumor-binding antibodies and adjuvants triggered both local and system-wide immune responses in this model, including lymph nodes, bone marrow and blood [87]. This could explain why the triple-negative breast cancer model is refractory to checkpoint blockade therapy, whereas the combination of alloIgG-IC with IFNγ and CD40 therapy is effective [87]. Binbin et al. developed a multimodal recurrent neural network called MARIA, which predicts the likelihood of antigen presentation for genes of interest in the context of specific HLA class II alleles. We propose that MARIA may be used to identify candidate antigens more accurately from patient sequencing data to improve alloIgG immunotherapy [88].

## AlloIgG immunotherapy may overcome current immunotherapy limitations

Immunotherapy has made considerable progress, with some patients achieving long-lasting responses through IgG McAbs and cellular immunotherapies. Therapeutic antibodies containing Fc domains promote antitumor activity by activating DCs [89]. Fc–FcγR interactions and uptake of ICs by DCs play a vital role in the in vivo activity of APCs and T cells through various mechanisms (Fig. 1) [90]. However, IgG McAb therapy often leads to drug resistance and tumor recurrence in 6 months via many different mechanisms [3]. We propose that alloIgG immunotherapy may overcome the limitations of IgG McAb therapy because it triggers robust systematic immune responses targeting multiple antigens via different signaling pathways (Figs. 2 and 3).

DC immunotherapy and vaccines have gained a crucial position due to their unique ability to present MHC class I and II molecule-restricted peptides and activate T cells (Fig. 1) [1, 91]. DCs are usually from the bone marrow or spleen and are rare. DC vaccines are feasible because DCs can now be cultured in large numbers ex vivo by controlling DC maturation and homing to lymph nodes [92]. A detailed protocol for isolating MoDCs from blood and tumors and activating MoDCs with tumor ICs is available [93]. There are two common methods to clinically prepare DC vaccines: loading tumor antigens directly onto DC cells or fusing DCs with tumor cells [94]. However, to date, DC-based vaccines have not achieved the expected therapeutic efficacy [95]. We propose that alloIgG immunotherapy or antigen-alloIgG complex-stimulated DC vaccines may cause the missing piece of the DC vaccine immunotherapy puzzle.

## The efficacy and safety perspectives of alloIgG immunotherapy

One limitation of alloIgG immunotherapy is that it works effectively only in tumors smaller than 20 mm^2^ in mouse models and becomes almost inert once the tumor exceeds an average size of approximately 40 mm^2^ due to tumor-infiltrating MoDC apoptosis [96]. One potential reason for this resistance might be the formation of cell-in-cell structures in large tumors [97]. Gutwillig et al*.* investigated the combination of dendritic cell adjuvants and tumor binding anti-TRP1 antibodies to treat a mouse model of relapse and found that the tumor cells remaining after immunotherapy form unique cell-in-cell structures and generate a membrane architecture that is impenetrable by immune-derived lytic granules, cytotoxic compounds, and chemotherapies. While reactive T cells can often kill the outer cells in this structure, the inner cells remain viable and intact, surviving for weeks in culture containing these T cells. Once the T cells are removed, the inner tumor cells disseminate back, suggesting that this biological process may be a central mechanism through which tumor cells evade T-cell immunity and give rise to relapsed tumors [97]. To increase the antitumor effects of alloIgG, we propose that alloIgG may be used in combination with other therapies, such as chemotherapy, radiotherapy, immune checkpoint inhibitors and CD4^+^ T cells. Several studies have shown that combining McAb IgG with conventional chemotherapy and radiotherapy can improve efficacy. For example, stereotactic body radiotherapy enhances the antitumor effects of the anti-PD-L1 McAb durvalumab in patients with early-stage NSCLC [98], and the combination of McAb pembrolizumab and radiotherapy has shown promising activity in patients with triple-negative breast cancer [99]. In patients with recurrent nasopharyngeal carcinoma, McAb toripalimab combined with intensity-modulated radiotherapy showed tolerability and promising antitumor activity [100]. Rasoulouniriana et al. discovered that CD4^+^ T cells isolated from tumors and tumor-binding antibodies have a strong synergistic effect to mediate tumor regression [101].

One of the safety concerns of alloIgG immunotherapy is whether it causes graft-versus-host disease (GVHD) due to genetic variation or polymorphisms among individual persons. AlloIgG immunotherapy exhibits therapeutic efficacy and safety in mouse models, although its promising therapeutic efficacy and safety in humans need to be tested [5, 64, 87, 96, 97, 101, 102]. Its prospective safety in humans may be further suggested by the safe use of allogeneic CAR-T cells, allogeneic γδT cells and natural killer cells [102–109]. Allogeneic γδT cells from haploidentical donors have been utilized to treat hematological malignancies, resulting in complete remission without signs of GVHD [105]. Furthermore, allogeneic Vγ9Vδ2 T-cell immunotherapy has demonstrated clinical safety and extended survival in patients with late-stage lung or liver cancer [107]. One advantage of alloIgG and allogeneic cell immunotherapy is that it allows for the preparation and storage of alloIgG and allogeneic cells in advance, thus reducing the waiting time and cost for patients to receive treatment [109].

## Review and view

The IgG Fc–DC FcγR interaction enables antigen recognition, processing and presentation by DCs, which activates T-cell immunity. Allogeneic tumors are eradicated by allogeneic IgG via DCs. AlloIgG combined with DC stimuli TNFα and CD40L induced tumor eradication via the reported and prospective signaling pathways. AlloIgG triggers systematic immune responses targeting multiple antigens, which was proposed to overcome current immunotherapy limitations. The promising efficacy and safety perspectives of alloIgG immunotherapy need to be validated.

With more efforts and breakthroughs, we believe that alloIgG tumor immunotherapy has promising potential to demonstrate efficiency and safety in mouse models, enter clinical trials and benefit tumor patients in the future. It has been 8 years since the first report of the important alloIgG tumor immunotherapy in *Nature* by Stanford University; however, there are only 6 directly related articles published mainly in mouse models (Table 1) [5, 64, 87, 96, 97, 101]. Therefore, we hope this perspective view of alloIgG tumor immunotherapy will provide an initiative to promote future discussion.

## Data Availability

Not applicable.

## Abbreviations

DCs: Dendritic cells
ICs: Immune complexes
alloIgG: Allogeneic IgG
IgG: Immunoglobulin G
FcγRs: Fcγ receptors
ADCC: Antibody-dependent cellular cytotoxicity
ADCP: Antibody-dependent cellular phagocytosis
moDC: Monocyte-derived DC
pDCs: Plasmacytoid DCs
mAbs: Monoclonal antibodies
APCs: Antigen-presenting cells
cDC1s: Type 1 cDCs
cDC2s: Type 2 cDCs
PAMPs: Pathogen-associated molecular patterns
PRRs: Pattern recognition receptors
imDCs: Immature DCs
TLRs: Toll-like receptors
FcRn: Neonatal Fc receptor
OVA: Ovalbumin
mAb-iFt: Nactivated *Francisella tularensis* immune complexes
TCR: T-cell receptor
MHC: Major histocompatibility complex
BMDCs: Bone marrow-derived dcs
TADCs: Tumor-associated dendritic cells
TNF-α: Tumor necrosis factor alpha
TRAFs: TNFR-associated factors
GP-NMB: Glycoprotein NMB
GVHD: Graft-versus-host disease
